# Quantitative fetal fibronectin to predict spontaneous preterm delivery after laser surgery for twin-twin transfusion syndrome

**DOI:** 10.1038/s41598-019-41163-8

**Published:** 2019-03-14

**Authors:** Andrew H. Chon, Yen Chan, Lisa M. Korst, Arlyn Llanes, Mira Abdel-Sattar, Ramen H. Chmait

**Affiliations:** 1 Division of Maternal-Fetal Medicine, Department of Obstetrics and Gynecology, Keck School of Medicine, University of Southern California, Los Angeles, California USA; 2 Childbirth Research Associates, North Hollywood, Los Angeles, California USA

## Abstract

Our goal was to assess whether quantitative fetal fibronectin (qfFN) is associated with spontaneous preterm birth (sPTB) after laser surgery for twin-twin transfusion syndrome (TTTS). qfFN was collected within 24 hours before and after laser surgery. Aims were: (1) To determine if qfFN changed with operative fetoscopy; and (2) To estimate the number of patients needed to study the predictive value of qfFN for sPTB <28 and <32 weeks. Results are reported as median (range). Among 49 patients, there was no net difference in qfFN levels after laser surgery [0.0 ng/mL (−37 to +400), p = 0.6041]. However, patients with a qfFN increase >10 ng/mL were 19 times more likely to undergo sPTB at <28 weeks (OR = 19.5). We determined that 383 and 160 patients would be needed to achieve adequate statistical power for qfFN to be predictive of sPTB at a GA <28 weeks and <32 weeks, respectively. In conclusion, laser surgery did not alter the qfFN level within the entire cohort, but qfFN may be useful in identifying a subset of patients at increased risk of preterm delivery.

## Introduction

Selective laser photocoagulation of communicating vessels (SLPCV) via fetoscopy is the preferred treatment for twin-twin transfusion syndrome (TTTS)^1^. However, the insertion of a trocar through the uterus and fetal membranes is considered a significant risk factor for preterm premature rupture of membranes (PPROM) and spontaneous preterm birth (sPTB)^2–5^. Strategies to predict and prevent prematurity in the field of fetal therapy are essential.

Biomarkers for singleton pregnancies such as fetal fibronectin (fFN), interleukin 6, C-reactive protein, placental alpha macroglobulin-1, and phosphorylated insulin-like growth factor binding protein-1 have been studied as potential predictors of sPTB^6–11^. Of these, fFN is the most well-studied. Fetal fibronectin is an extracellular matrix protein acting as a physiologic “glue” at the decidual-chorionic junction^12^. Disruption of this interface due to processes such as uterine contractions or localized inflammation at the choriodecidual interface leads to release of fFN into cervicovaginal secretions.

Despite the advances in biochemical tests in predicting sPTB in singletons, the reliability of such markers remains controversial in twin gestations. Cervical length and fFN have been most commonly studied in twin gestations and the results have been mixed^13–17^. Some studies including Matthews *et al*.^16^. and Fuchs *et al*.^17^. have shown an association with a positive fFN result and sPTB in asymptomatic and symptomatic twin pregnancies, respectively. The literature is even more limited regarding predictors of sPTB for TTTS patients. The aims of this study were: (1) To determine if quantitative fFN (qfFN) changed significantly with operative fetoscopy; and (2) To estimate the number of patients needed to study the predictive value of qfFN for sPTB occurring <28 and <32 gestational weeks in these patients.

## Methods

This was a prospective observational cohort study of all mothers with consecutive monochorionic diamniotic twin gestations who underwent laser surgery for TTTS from July 2015 to January 2017 at Los Angeles Fetal Surgery (University of Southern California). Each patient underwent a comprehensive ultrasound examination preoperatively and on postoperative day 1, which consisted of a detailed anatomic survey, measurement of the amniotic fluid maximum vertical pocket (MVP) for each twin, fetal Doppler assessments of the umbilical artery, umbilical vein, ductus venosus and middle cerebral artery, as well as cervical length. TTTS was diagnosed if the monochorionic diamniotic twin gestation had a MVP of fluid ≥8 cm in the recipient twin’s sac and ≤2 cm in the donor’s sac. The patients were staged prospectively according to the Quintero staging system^18^. Cases were treated exclusively by SLPCV with or without sequential technique, as described in detail previously^19,20^. Study inclusion criteria were twin gestations with TTTS who were treated with laser surgery at a gestational age (GA) of 16–26 weeks. Exclusion criteria included receiving a cerclage during the pregnancy, dual intrauterine fetal demise, vaginal penetration within 24 hours of fFN collection, cervical dilation >3 cm, frank vaginal bleeding, or rupture of membranes (prior to pre- or postoperative qfFN collection). At our center, patients with a CL ≤2.0 cm were offered cerclage placement at the time of laser surgery^21,22^.

Each patient underwent a pre- and postoperative fFN collection prior to endovaginal cervical length measurement. Preoperative collections were done within 24 hours of surgery. Postoperative collections were performed at least 24 hours after surgery on postoperative day 1. The Hologic Rapid fFN^®^ Test Specimen Collection Kit and 10Q System were used per manufacturer recommendations to determine the quantitative fFN (qfFN) concentrations.

The acquisition and testing of the qfFN was standardized and was in compliance with the Human Subject Research regulations of University of Southern California. In brief, a speculum exam without gel lubrication was performed to visualize the cervix and posterior vaginal fornix. The sterile swab was lightly rotated across the posterior fornix for approximately 10 seconds to absorb cervicovaginal secretions. Specimens were stored at room temperature and processed within 8 hours using the Rapid fFN 10Q Analyzer. The cervicovaginal specimen was immersed in buffer and a 200 µL sample was pipetted into the well of the Rapid fFN 10Q Cassette. The qfFN concentration reported by the analyzer (range 0 to 500 ng/mL) was prospectively recorded. Concentrations greater than 500 ng/mL were displayed as >500 ng/mL. After laser surgery, the patients spent 1 night in the hospital, and were placed on tocolysis only if clinically significant contractions developed. Postoperative ultrasound was performed on postoperative day 1 and patients returned to their referring perinatologist to be managed for the remainder of the pregnancy. The study investigators and treating physicians were blinded to all of the qfFN results.

Information that was gathered and prospectively recorded in a database included: maternal demographics, pre- and postoperative gestational findings, surgical characteristics, and delivery information. The delivery indication was categorized as spontaneous labor or non-spontaneous. Spontaneous labor was defined as regular painful uterine contractions accompanied by cervical change, with or without ruptured fetal membranes. Spontaneous labor included patients with PPROM because risk factors for PPROM are generally similar to those for preterm spontaneous labor with intact membranes, and because most patients with PPROM begin labor spontaneously within several days^23^. No distinction was made between iatrogenic and spontaneous PPROM.

The study aims were: (1) To determine if qfFN changed significantly within 24-hours of operative fetoscopy and laser surgery for TTTS; and (2) To estimate the number of patients needed to study the predictive value of qfFN for sPTB occurring <28 and <32 gestational weeks. Patient characteristics and outcome data were initially evaluated univariately, and for study aim (1), the distribution of change in qfFN values was tested for difference from zero using a one-sample Wilcoxon signed rank test. Bivariate statistical analyses were then performed to compare patients with and without a qfFN increase >10 ng/mL after laser surgery to identify any patient characteristics associated with such an increase (p < 0.15). A difference of >10 ng/mL was arbitrarily chosen to represent the minimal clinically relevant increase in qfFN. In studies performed by the manufacturer (https://www.hologic.com/sites/default/files/package-insert/AW-09189-002_004_02.pdf), the quantitative measurement of the fFN level was fairly precise, with a standard deviation of 3.2 and a coefficient of variation of 6–8%. Hence, in particular, at lower fFN concentrations, the absolute variation of interest (>10 ng/mL) is outside this measurement variation and the results of our analyses regarding the differences between fFN measurements should be reliable. Bivariate analyses of continuous variables were performed with Kruskal-Wallis testing; bivariate analyses of categorical variables were performed with Fisher exact testing. Eligible patient characteristics were then tested as covariates in multiple logistic regression models to identify any significant relationship with a pre- to postoperative change in qfFN >10 ng/mL and multiple linear regression models to identify any significant relationship with a pre-to postoperative change in qfFN.

For study aim (2), we used bivariate analyses to test our predictor variables, increase in qfFN (>10 ng/mL) and a postoperative qfFN >50 ng/mL, versus sPTB occurring <28 and <32 gestational weeks. A postoperative qfFN increase of ≥50 ng/mL was chosen because the Rapid fFN^®^ is considered ‘positive’ with concentrations at least 50 ng/mL. We also used bivariate analyses to identify predictors of these same sPTB outcomes to select potential covariates for multiple logistic regression models testing the relationship between qfFN levels and sPTB. Results are expressed as n (%) or median (range). Odds ratios (OR) are reported with 95% confidence intervals (95% CI). All analyses were performed using SAS statistical software (Version 9.2, SAS Institute Inc., Cary, NC). The study was conducted with an investigator-initiated unrestricted grant. Hologic® supplied all necessary materials including the Rapid fFN® Test Specimen Collection Kit and 10Q System, and played no role in the conduct of the study. Informed written consent was obtained from all patients. Consent was provided by the patient and the parent or legal guardian for patients under the age of 18 years old. This study and the experimental protocol was approved by the Institutional Review Board of the University of Southern California, and complied with all patient protection criteria stipulated therein. All the methods and experiments utilized in the study were in accordance with the guidelines and regulations of the Health Sciences Institutional Review Board.

## Results

Of the 93 patients who underwent laser surgery, 49 (52.7%) met study criteria. The reasons for exclusion of the remaining 44 patients are listed in Table 1. Among the 40 excluded twin gestations, 38 underwent laser surgery at 20.3 (16.4–26.4) weeks and delivered at 34.2 (18.4–37.7) weeks. Two twin gestations did not undergo laser surgery because one had close proximity of the placental cord insertions and another had recipient twin demise (TTTS stage V) the morning of surgery. Of the 9 patients with cerclage during the pregnancy, 4 were placed prior to laser surgery (3 by the referring provider), 2 on the same day as surgery, and 3 in the postoperative period. The GA at delivery was 32.0 (20.0–36.6) weeks among patients with cerclage placement. Two of the 49 included patients had a history of a prior spontaneous preterm birth. Nearly all included patients had some degree of maternal discomfort secondary to polyhydramnios and abdominal distention. Two patients had clinically significant subjective contractions, although both had a preoperative cervical length greater than 3.5 cm. The median GA at surgery was 19.1 (17.3–24.3) weeks and at delivery was 33.1 (23.7–38.3) weeks. PPROM occurred in 9 patients at 9.3 (0.9–16.9) weeks.

**Table 1:** Reasons for exclusion from qfFN study.

Reason for exclusion	Number of patients
Cerclage placement during pregnancy	9
Vaginal penetration 24 hours prior to preop	7
Patient declined	6
qfFN collection not performed	6
qfFN sample not tested within 8 hours	5
Triplet gestation	4
Laser surgery beyond 26 weeks	2
PPROM prior to postoperative qfFN collection	2
Frank vaginal bleeding	1
Dual intrauterine fetal demise	1
Invalid qfFN reading	1

qfFN, quantiative fetal fibronectin; PPROM, preterm premature rupture of membranes.

**Figure 1:**
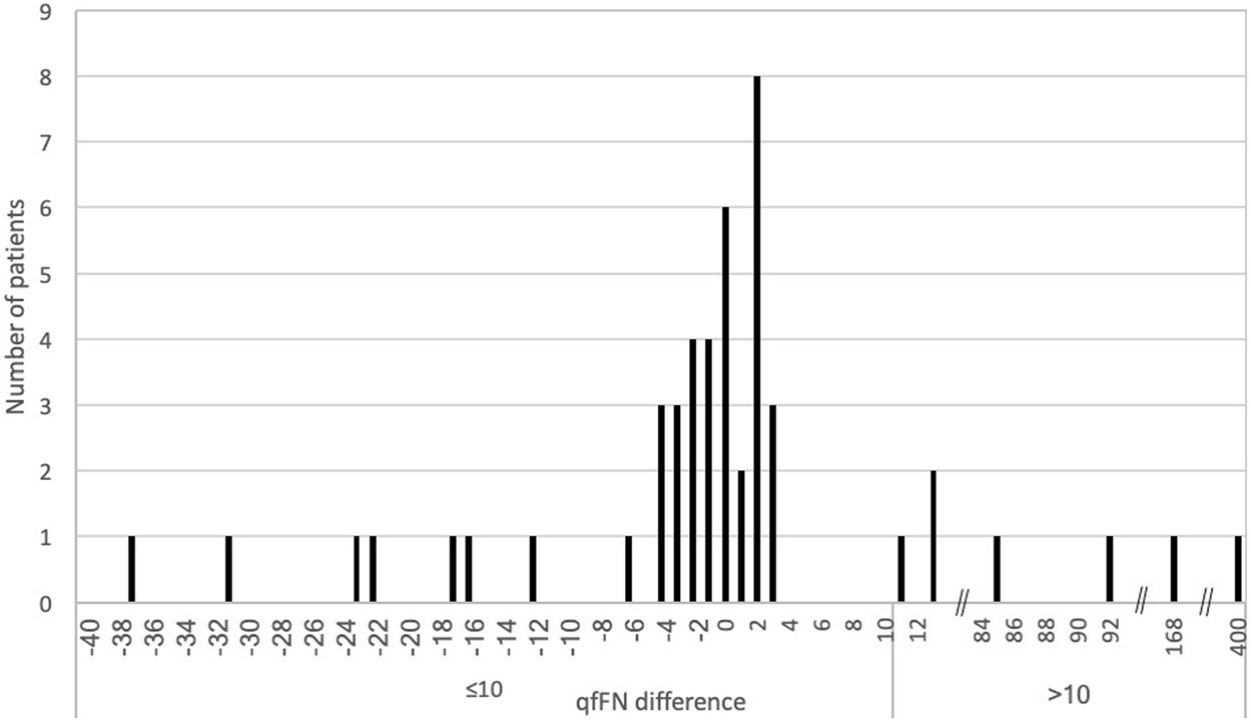
Distribution of net qfFN difference (ng/mL) after laser surgery for twin-twin transfusion syndrome.

There was no significant net difference in qfFN after laser surgery in the entire cohort [median 0.0 ng/mL (−37 to +400), p = 0.6041]. The numbers of patients with and without a >10 ng/mL postoperative difference in qfFN were 7 (14.3%) and 41 (83.7%), respectively. (Fig. 1, Table 2) Among the 7 patients with a postoperative difference in qfFN >10 ng/mL, the median interval from surgery to delivery was 65 (19–76) days, with a median delivery GA of 28.0 (23.7–35.0) weeks. The indications for delivery were: spontaneous preterm labor (n = 6) and placental abruption (n = 1). An increase >10 ng/mL in qfFN after laser surgery was also associated with an earlier GA at delivery and a correspondingly longer neonatal intensive care unit (NICU) admission (Table 2).

**Table 2:** Pregnancy characteristics of patients who underwent laser surgery and pre- and postoperative qfFN collection.

Patient characteristic	Patients with qfFN difference >10 ng/mL (N = 7)	Patients with qfFN difference ≤10 ng/mL (N = 42)	P value
Multiparous	3 (42.9%)	26 (61.9%)	0.4221
History of sPTB	0 (0%)	2 (4.8%)	1.0000
Quintero stage 3 or 4	4 (57.1%)	29 (69.0%)	0.6685
Preoperative membrane detachment	2 (28.6%)	14 (33.3%)	1.0000
GA at surgery	20.1 (18.0–24.1)	19.1 (17.3–24.4)	0.6066
Preoperative cervical length <3.5 cm	4 (57.1%)	10 (23.8%)	0.0914
Preoperative cervical length (cm)	3.27 (3.06–4.41)	3.99 (2.63–5.29)	0.0383
Preoperative EFW percentile recipient	84.1 (34.7–99.0)	60.2 (4.6–99.0)	0.1096
Preoperative qfFN	52.0 (4.0–433.0)	4.0 (1.0–93.0)	0.0058
Preoperative qfFN high (≥50 ng/mL)	4 (57.1%)	1 (2.4%)	0.0008
Postoperative qfFN	155.0 (15.0–446.0)	3.0 (0.0–56.0)	<0.0001
Postoperative qfFN high (≥50 ng/mL)	5 (71.4%)	1 (2.4%)	<0.0001
Postoperative cervical length (cm)	2.98 (2.04–4.49)	4.12 (2.81–6.12)	0.0022
Postoperative membrane detachment	2 (28.6%)	14 (33.3%)	1.0000
Postoperative tocolysis	2 (28.6%)	2 (4.8%)	0.0924
Interval from surgery to delivery (days)	65.0 (19–76)	102.0 (42–131)	0.0051
Delivery GA	28.0 (23.7–35.0)	33.9 (26.4–38.3)	0.0082
NICU days of donor	n = 6 44.0 (21–118)	n = 39 16.0 (0–91)	0.0319
NICU days of recipient	n = 7 64.0 (30–114)	n = 39 17.0 (0–87)	0.0045

qfFN, quantitative fetal fibronectin (ng/mL); sPTB, spontaneous preterm birth; GA, gestational age; EFW, estimated fetal weight; NICU, neonatal intensive care unit.

Furthermore, an increase >10 ng/mL was associated with both a higher preoperative and postoperative qfFN concentration. Trocar placement was not a risk factor for the qfFN increase in this population. The number of patients with trocar insertion in the lower uterine segment in patients with and without a >10 ng/mL difference were 1 (14.3%) and 4 (9.5%) p = 0.5539, respectively. In multiple logistic regression models testing only preoperative potential risk factors identified in bivariate analyses (preoperative qfFN ≥50 ng/mL, cervical length, donor/recipient velamentous insertion, and estimated fetal weight percentile of the recipient), only a preoperative qfFN ≥50 ng/mL was associated with a pre- to postoperative change in qfFN >10 ng/mL (OR = 54.65 [4.55–655.85], p = 0.0016). No potential risk factors were associated with qfFN in multiple linear regression.

Table 3 displays characteristics of patients who underwent sPTB <28 gestational weeks and <32 gestational weeks. sPTB <28 gestational weeks occurred in 3 patients (6%), 2 of which had a >10 ng/mL increase in qfFN after surgery. In bivariate analysis, patients with a qfFN increase >10 ng/mL were 19 times more likely to undergo sPTB <28 gestational weeks (OR = 19.5, 95% CI 1.4–265.6; p = 0.0258). In bivariate analysis, patients with a postoperative qfFN ≥50 ng/mL appeared to be 6 times more likely to undergo sPTB <28 gestational weeks (OR 6.6, 95% CI 0.7–61.8, p = 0.1003, non-significant). Neither pre- or postoperative cervical length was significantly associated with sPTB <28 gestational weeks. Multiple logistic regression analysis was not feasible due to the small number of patients with the sPTB <28 weeks outcome.

**Table 3:** Comparison of patients with sPTB <28 weeks and <32 weeks.

Patient characteristic	sPTB <28 wks (N = 3)	sPTB ≥28 wks (N = 43)	sPTB <32 wks (N = 14)	sPTB ≥32 wks (N = 31)
Multiparous	2 (66.7%)	24 (55.8%)	10 (71.4%)	15 (48.4%)
History of sPTB	0 (0%)	1 (2.3%)	0 (0%)	1 (3.2%)
Quintero stage 3 or 4	2 (66.7%)	28 (65.1%)	7 (50.0%)	22 (71.0%)
GA at surgery	18.3 (18.0–23.7)	19.1 (17.3–24.4)	20.2 (18.0–24.4)	19.1 (17.3–24.1)
Preoperative CL <3.5 cm	1 (33.3%)	12 (27.9%)	7 (50.0%)	6 (19.4%)
Preoperative qfFN	70.0 (3.0–433.0)	4.0 (1.0–93.0)	14.5 (1.0–433.0)	4.0 (1–93)
qfFN preoperative high (≥50)	2 (66.7%)*	3 (7.0%)	3 (21.4%)	2 (6.5%0)
Postoperative qfFN	155.0 (6.0–446.0)	4.0 (0–404)	7.5 (0.0–446.0)*	3.0 (1–404)
qfFN postoperative high (≥50)	2 (66.7%)*	4 (9.3%)	3 (21.4%)	3 (9.7%)
Postoperative tocolysis	0 (0%)	4 (9.3%)	0 (0%)	4 (12.9%)
qfFN net difference	13.0 (3.0–85.0)*	−1.0 (−37 to +400)	1.0 (−31 to +168)	−1.0 (−37 to +400)

*P value < 0.05 for comparison of sPTB <28 weeks with ≥28 weeks, or sPTB <32 weeks with ≥32 weeks.

sPTB, spontaneous preterm birth; GA, gestational age; qfFN, quantitative fetal fibronectin (ng/mL); CL, cervical length.

sPTB <32 gestational weeks occurred in 14 patients (29%). In bivariate analysis, patients with a qfFN increase >10 ng/mL appeared nearly 6 times more likely to undergo sPTB <32 gestational weeks (OR = 5.8, 95% CI 0.9–36.6, p = 0.0616, non-significant). In bivariate analysis, patients with a postoperative qfFN ≥50 ng/mL appeared to be more likely to undergo sPTB <32 gestational weeks (OR 2.5, 95% CI 0.4–14.6, p = 0.2942, non-significant). Patients with a greater postoperative cervical length were less likely to undergo sPTB <32 gestational weeks (OR 0.18, 95% CI 0.04–0.8, p = 0.0235). As with the <28 gestational weeks outcome, multiple logistic regression analysis was not feasible due to the small number of patients.

Power analyses were conducted, determining that 383 patients would be needed to achieve adequate statistical power for qfFN to be predictive of sPTB <28 gestational weeks. A logistic regression of a binary response variable (i.e., sPTB <28 gestational weeks) on a binary independent variable (i.e., 10 ng/mL increase in qfFN) with a sample size of 383 observations (of which 94% are in the group where sPTB <28 weeks = no and 6% are in the group where sPTB <28 weeks = yes) achieves 80% power at a 0.05 significance level to detect a change in probability (y = 1) from the baseline value of 0.750 to 0.983. This change corresponds to an OR of 19.50. An adjustment was made since a multiple regression of the independent variable of interest on several other independent variables potentially predictive of sPTB <28 gestational weeks in the logistic regression was hypothesized to obtain an R-square of 0.200. These other potential independent variables were identified in bivariate analyses with sPTB <28 gestational weeks (e.g., cervical length, laser time, GA at surgery, Quintero stage, and donor intrauterine growth restriction).

Similar calculations yielded a sample size of 160 observations required for testing of the prediction of sPTB <32 weeks from a 10 ng/mL increase in qfFN, given a similar contribution of other independent variables, an OR of 5.80 and 69% of the sample with PTB <32 weeks = no.

## Discussion

The current study investigated the immediate postoperative changes in qfFN concentration as a result of operative fetoscopy and laser surgery for TTTS. This minimally invasive procedure involves insertion of a trocar into the recipient twin’s sac. The trocar disrupts the interface between the decidua and fetal membranes and presumably can lead to disruption of the decidual-chorionic interface, thereby releasing fetal fibronectin into the cervico-vaginal secretions. Our study demonstrated in Aim 1 that operative fetoscopy did not appear to be associated with a difference between the pre- and postoperative qfFN concentration. These results may largely be attributed to the relatively focal disruption of the decidual-chorionic junction by the trocar. The combination of the small size of the defect and its remote location away from the posterior vaginal fornix may have limited the amount of qfFN in the cervico-vaginal secretions within 24 hours after surgery.

For Aim 2, to estimate the number of patients needed to study the predictive value of qfFN with respect to sPTB, we found that 383 and 160 patients would be needed to achieve adequate statistical power for qfFN to be predictive of sPTB at a GA <28 weeks and <32 weeks, respectively, controlling for other potential predictors of sPTB. This was based on our findings that suggest that patients who had a >10 ng/mL increase in qfFN after surgery may be at an increased risk of sPTB. The median delivery GA in this subset was 28.0 gestational weeks compared to 33.9 gestational weeks in patients with ≤10 ng/mL difference. Our sample size was too small to investigate this further, though larger future studies may be able to explore and confirm these initial findings.

Strengths of the study include prospective design, uniform surgical technique at an experienced fetal surgery center, simultaneous measurement of both cervical length and qfFN, and blinding of the investigators to the qfFN results. Furthermore, all referring providers were unaware of the qfFN results and hence the results did not alter pregnancy management. All measures of endovaginal cervical length were performed by a single experienced sonographer.

The study has several limitations. First, the number of patients with sPTB <28 and <32 gestational weeks was small and thus limited our ability to control for covariates. A contributing factor to small patient numbers was that only approximately 50% of eligible candidates were enrolled. Among the excluded were 9 patients with a cerclage. Although the presence of a cerclage was an exclusion criterion for this study as it would be a confounding variable in the prediction of sPTB, to eliminate these patients simultaneously eliminated the subset of patients at highest risk of spontaneous preterm birth. To further study this high-risk subset of patients, we propose a future study comparing serial qfFN levels in TTTS patients with and without a cerclage. In addition, the current study did not demonstrate preoperative cervical length to be a significant risk factor for preterm birth, However, prior larger studies have demonstrated preoperative cervical length to be an important predictor of preterm birth after laser surgery for TTTS^2,4,24^. For instance, thresholds such as a preoperative cervical length <20 mm^2^ or <28 mm^24^ have been associated with an increased risk spontaneous preterm birth after laser surgery. Furthermore, as our power analysis indicates, and given the multiple risk factors for sPTB, a much larger study with nearly 400 patients would be needed to adequately detect a significant effect size in sPTB <28 weeks.

Second, due to the acute nature of the TTTS, patients are often evaluated at our center within 24 hours of the referring perinatologist having performed the initial ultrasound assessment, which often includes an endovaginal cervical length measurement. This makes a fair number of patients ineligible for qfFN collection prior to evaluation at our center. Furthermore, serial postoperative qfFN measurements were not performed, preventing us from assessing the temporal relationship to operative fetoscopy. Third, previous studies assessing the reliability of fFN to predict preterm birth have separated patients with and without symptoms of labor^16,25–27^. A pilot study by Fuchs *et al*. showed a potential application of qualitative fFN in twin gestations complicated by preterm labor^17^. Although the fFN test was positive in only 3 (7.5%) patients, it seemed to predict spontaneous preterm birth within 7 days better than cervical length^17^. Although only 2 patients in our study complained of significant uterine contractions, TTTS patients commonly have abdominal discomfort from polyhydramnios. Since routine preoperative external tocodynamometry was not performed, we are unable to precisely determine the number of patients with regular uterine contractions. Hence, both Fuchs *et al*.^17^ and our study highlight different subsets of twin gestations in which a bedside fFN test could alter the counseling of patients in regards to the risk of preterm delivery.

PPROM was included as part of the definition of spontaneous labor because of its close association with preterm labor. In addition, although the pathophysiology is different in surgical iatrogenic PPROM (iPPROM) compared to spontaneous PPROM, we did not make a distinction between these groups for two reasons; (1) there is no agreed upon definition for iPPROM, and (2) iPPROM has been previously associated with spontaneous labor^4,28^. Given the median interval from surgery to PPROM was 9.3 weeks and only one patient had PPROM within 7 days of surgery, the number of patients with iPPROM is likely few. Fourth, we did not measure qfFN in uncomplicated monochorionic diamniotic twin pregnancies, hence do not have validated reference ranges available for comparison. Lastly, the Rapid fFN 10Q Analyzer (Hologic) is not currently commercially available in the United States and is still considered investigational. Although qfFN measurement using the Hologic Rapid Analyzer is a low-cost bedside test, its clinical utility remains in question.

To our knowledge, this is the first study investigating the use of qfFN after an invasive *in utero* procedure. There appears to be potential for identifying patients at increased risk of preterm delivery <28 weeks by using qfFN. The results of our study pertain to a specific group of patients, that is, monochorionic diamniotic twins without a cerclage treated by laser surgery for TTTS. A larger study with this target patient population would add clarity to the clinical utility of perioperative use of qfFN measurements in predicting spontaneous preterm birth. Until then, the results of the current study should be considered preliminary and interpreted with caution given the aforementioned limitations. Another potential area of research is evaluating the predictive value of a single preoperative qfFN value, which was not the focus of the current study. Abbott *et al*. reported that in patients with singleton pregnancies and symptoms of preterm labor, the positive predictive value for sPTB <34 weeks increased from 19%, 32%, 61%, and 75% with increasing thresholds (10, 50, 200, and 500 ng/mL, respectively)^29^. In our study, preoperative qfFN ≥50 ng/mL was associated with a net qfFN increase of >10 ng/mL, and perhaps this subset of TTTS patients is at higher risk of sPTB. Future larger studies will help determine the utility of qfFN in TTTS patients treated with laser surgery and identify clinically useful thresholds for identifying patients at particularly high risk of sPTB.
